# Managing the consequences of rising mental‐health awareness—a commentary on Marcotulli et al. (2025)

**DOI:** 10.1111/jcpp.70195

**Published:** 2026-06-18

**Authors:** Matthias Pierce

**Affiliations:** ^1^ Division of Psychology and Mental Health, Centre for Women's Mental Health, Faculty of Biology, Medicine and Health University of Manchester Manchester UK

**Keywords:** Mental health

## Abstract

The editorial by Marcotulli, Foulkes and Stringaris presents a dynamic‐systems framework for understanding the effects of mental‐health information campaigns. They argue that surges in help‐seeking may lead to poorer diagnostic accuracy and reduced treatment efficacy, with potential harm to patients. Their systems‐level approach is timely and valuable. The editorial's predictions rest on several assumptions: new help‐seekers have lower severity, diagnostic thresholds drift downward and the benefit–harm ratio of treatment declines. Empirical evidence supporting these assumptions remains limited. Where these mechanisms do operate, several strategies may mitigate their impact. This commentary outlines key assumptions requiring empirical testing and discusses mitigation approaches, including targeted campaigns and effective triage systems, which may reduce the risk that increased awareness leads to net harm.

## Introduction

The editorial by Marcotulli, Foulkes, and Stringaris (2025) presents a framework for understanding how mental‐health information campaigns can lead to negative consequences. Their dynamic models show how rising awareness can trigger a sequence of processes that produce unintended negative consequences. This perspective is timely in young people's mental health, given the substantial increase in help‐seeking and the current strain on services (Children's Commissioner for England, 2025). The editorial illustrates how greater awareness, resulting in rising help‐seeking, can lower the prevalence of genuine cases within services, restrict access for those who need help and reduce the average benefit amongst those receiving treatment. Many of these predicted harms stem from shifts in the diagnostic process, including more false positives as lower‐prevalence populations present for assessment and missed true cases when demand outstrips capacity.

These are important potential consequences to highlight, but they rest on specific assumptions about how clinicians and health systems respond to greater awareness and rising demand. Robust empirical assessment of these assumptions will help to ratify conclusions; however, where they do hold, strategies are available to mitigate their effects. Many of these mitigation strategies are likely already deployed, either formally or informally, meaning the consequences of increased awareness may not be as negative as predicted. This commentary highlights some key assumptions underlying the editorial's cautionary conclusions and provides an overview of mitigation strategies that can limit adverse consequences of increased mental‐health awareness.

## Assumption 1: New help‐seekers are lower risk

The editorial assumes that greater awareness results in more individuals with milder symptoms seeking help, reducing the overall prevalence in help‐seekers. As prevalence amongst help‐seekers falls, the proportion of diagnosed individuals who are genuine cases (the positive predictive value) also falls. This downward shift in severity is very plausible, although it is more likely in some diagnostic domains (e.g. autism; see Russell et al., 2022) than in others (e.g. psychosis).

### Mitigation strategies

- *Targeting campaign efforts towards groups with low uptake of services*. Mental‐health information campaigns generally aim to encourage timely help‐seeking. The downward drift in severity amongst new help‐seekers may be mitigated if campaigns effectively target groups with historically low uptake of services. For example, minority ethnic groups in the UK are least likely to access mental‐health support in primary care yet are at higher risk of compulsory detention (Bignall, Jeraj, Helsby, & Butt, 2020). Encouraging timely access amongst low‐access groups is likely to bring in individuals with prevalence comparable to, or higher than, those already seeking support, meaning the prevalence amongst help‐seekers does not fall.
- *Campaigns targeted at improving identification of mental‐health symptoms*. Mental‐health information campaigns could offset potential harms by helping individuals identify when professional support is warranted. For example, future campaigns may be designed to help patients recognise persistent or impairing symptoms relative to transient distress.
- *Using initial screening to fast‐track high‐risk help‐seekers to full assessment*. Brief initial screening can be used to stratify help‐seekers according to probable severity and risk. Individuals with high symptom severity and functional impairment can be prioritised for full specialist assessment. If increased awareness leads to larger numbers of individuals endorsing symptoms, structured triage systems may help preserve timely access for those at greatest clinical need and stabilise the case‐mix entering specialist services.

## Assumption 2: Diagnostic thresholds drift downward with increasing help‐seeking

In the editorial's model, clinicians are influenced by greater awareness and social pressures to lower their threshold for diagnosis, which reduces specificity and lowers the positive predictive value. This is theoretically plausible; however, quantification of the extent to which increased awareness (rather than changes in nosology) changes diagnostic boundaries is unclear.

### Potential mitigation strategy

- *Ongoing monitoring of diagnostic performance*. Regularly calculating measures of diagnostic performance can provide services with timely feedback on whether thresholds are drifting. Routine service data can be used for this purpose, for example calculating the proportion of those who were diagnosed who were subsequently rapidly discharged, potentially suggesting that treatment was unwarranted, although discharge reasons are likely multifactorial. The metrics that are appropriate for monitoring performance will likely vary by condition, for example what works for autism may not work for psychosis. Feeding this information back to diagnosticians may prompt recalibration of thresholds, helping maintain diagnostic accuracy.

## Assumption 3: New, milder cases in services receive less benefit from treatment while being equally exposed to harms

The editorial suggests that treatment may be less effective for milder cases that are drawn to services through greater awareness, while the risk of adverse side‐effects remains similar across severity strata. As a result, the proportion who benefit from treatment reduces, whereas the number harmed per treated patient remains largely unchanged. This combination reduces the overall net benefit of treatment. The extent that this is true likely varies by disorder and intervention type. In some instances, treatment may be less effective amongst severe cases, who may have more intractable, hard‐to‐treat conditions.

### Potential mitigation strategies

- *Earlier identification*. Greater awareness may lead individuals, or those close to them, to recognise symptoms earlier in the course of a disorder. For some conditions, earlier intervention is associated with better long‐term outcomes (for example, Kraus, Kadriu, Lanzenberger, Zarate, & Kasper, 2019). Earlier, effective intervention would improve outcomes for patients and decrease burden on services as a result of long‐term need.
- *Personalised treatment pathways*. Following diagnosis, patients are matched to treatments that are considered most suitable. This may mean that lower‐severity cases receive lower‐intensity interventions with low risk of adverse effects (e.g. brief psychoeducation), while reserving specialist treatments (e.g. psychotropic medications) for high‐risk patients. This personalised approach reduces exposure to harm amongst milder cases and protects the overall benefit–harm balance.

## Next steps

The editorial calls for more testing of the impact of mental‐health information campaigns. This would be useful. However, mental‐health awareness has spread beyond formal campaigns and into public consciousness (facilitated by social media). Therefore, the extent that it can be easily reversed is uncertain. What is needed are clear approaches to best deal with the large number now seeking help.

A first step is to quantify changes in severity amongst help‐seekers and diagnosed patients. This may involve repeated surveys, provided measurement properties have not also shifted with changing awareness (Ahuvia, 2024). Alternatively, it may involve examining changes in the functional aspects of mental disorder, such as school dismissal or hospital attendances for acute distress. A second step is to map which strategies are currently used by referrers and mental‐health services to adjust to increasing demand; investigate their effectiveness, aligned to system impacts; and highlight best practice. Where gaps are identified, implementation of strategies should follow, followed by robust testing of their impacts.

## Conclusion

Young people's mental‐health services are experiencing unprecedented levels of demand. Much of this increase likely comes from more awareness. Whether these trends can be reversed, or will shortly plateau, is unclear. The editorial's dynamic‐systems approach is a helpful framework for understanding what this has meant for patients, but conclusions of harm require more empirical testing of assumptions and a clear understanding of the use and consequences of mitigation strategies. Implementing these strategies may require resources, for example training primary‐care professionals to improve detection, but overall could improve patient outcomes and lower costs associated with a poorly performing system.

## Data Availability

Data sharing not applicable to this article as no datasets were generated or analysed during the current study.
